# Epithelial Keratins Modulate cMet Expression and Signaling and Promote InlB-Mediated *Listeria monocytogenes* Infection of HeLa Cells

**DOI:** 10.3389/fcimb.2018.00146

**Published:** 2018-05-14

**Authors:** Rui Cruz, Isabel Pereira-Castro, Maria T. Almeida, Alexandra Moreira, Didier Cabanes, Sandra Sousa

**Affiliations:** ^1^Group of Molecular Microbiology, Instituto de Investigação e Inovação em Saúde, Universidade do Porto, Porto, Portugal; ^2^Group of Molecular Microbiology, Institute for Molecular and Cell Biology, Porto, Portugal; ^3^Instituto de Ciências Biomédicas Abel Salazar, Universidade do Porto, Porto, Portugal; ^4^Gene Regulation Group, Institute for Molecular and Cell Biology, Porto, Portugal

**Keywords:** intermediate filaments, keratins, cMet signaling, *Listeria monocytogenes*, cellular infection, mRNA stability, gene expression

## Abstract

The host cytoskeleton is a major target for bacterial pathogens during infection. In particular, pathogens usurp the actin cytoskeleton function to strongly adhere to the host cell surface, to induce plasma membrane remodeling allowing invasion and to spread from cell to cell and disseminate to the whole organism. Keratins are cytoskeletal proteins that are the major components of intermediate filaments in epithelial cells however, their role in bacterial infection has been disregarded. Here we investigate the role of the major epithelial keratins, keratins 8 and 18 (K8 and K18), in the cellular infection by *Listeria monocytogenes*. We found that K8 and K18 are required for successful InlB/cMet-dependent *L. monocytogenes* infection, but are dispensable for InlA/E-cadherin-mediated invasion. Both K8 and K18 accumulate at InlB-mediated internalization sites following actin recruitment and modulate actin dynamics at those sites. We also reveal the key role of K8 and K18 in HGF-induced signaling which occurs downstream the activation of cMet. Strikingly, we show here that K18, and at a less extent K8, controls the expression of cMet and other surface receptors such TfR and integrin β1, by promoting the stability of their corresponding transcripts. Together, our results reveal novel functions for major epithelial keratins in the modulation of actin dynamics at the bacterial entry sites and in the control of surface receptors mRNA stability and expression.

## Introduction

Intracellular pathogens exploit the host machinery to promote and establish infection. The host cytoskeleton is one of the preferential targets of pathogens and plays essential roles in cellular infection (Carabeo, 2011; Haglund and Welch, 2011; de Souza Santos and Orth, 2015). The role of host actin cytoskeleton in bacterial pathogenesis is by far the most documented (Colonne et al., 2016). Actin filaments and their polymerization machinery are hijacked by several human pathogens at different stages of the infection process. In particular subversion of actin is critical for: (1) stable adhesion of pathogenic *Escherichia coli* (EPEC and EHEC) to the host cell surface, through the formation of actin-rich pedestals (Goosney et al., 2000; Gruenheid et al., 2001; Stradal and Costa, 2017); (2) invasion of epithelial cells by a variety of intracellular bacteria such as *Salmonella typhimurium, Shigella flexneri*, and *Listeria monocytogenes* which induce actin cytoskeleton rearrangements and host membrane remodeling (Bierne et al., 2005; Sousa et al., 2007; de Souza Santos and Orth, 2015; Valencia-Gallardo et al., 2015; Rolhion and Cossart, 2017); and 3) intracellular movement of cytosolic pathogens such as *S. flexneri, Rickettsia conorii*, and *L. monocytogenes* which are able to elicit the formation of actin comet tails to promote cell-to-cell spread (Bernardini et al., 1989; Mounier et al., 1990; Welch et al., 1997; Egile et al., 1999; Heinzen et al., 1999; Czuczman et al., 2014; Kuehl et al., 2015).

In contrast to actin, the role of intermediate filaments (IFs), in particular keratins, during bacterial infection is poorly characterized. IFs are also part of the host cytoskeleton and include a large group of proteins that share structural features and form apolar 10 nM wide fibrous filaments (Goldman et al., 2012). Keratins are the largest subfamily of IFs, mainly expressed in the cytoplasm of epithelial cells and their expression profile is regulated in a tissue and differentiation dependent manner (Loschke et al., 2015). Type I and type II keratins form heterodimers and organize into filaments that ensure structural integrity of epithelia and confers mechanical resilience to stress (Haines and Lane, 2012). In simple epithelial cells, Keratin 8 (K8) and Keratin 18 (K18) are the most common keratin pair (Moll et al., 2008). Besides their biomechanical functions, several studies point keratins as important players in regulatory mechanisms defining health and disease (Pan et al., 2012). K8 and K18 participate in cell cycle regulation by associating with and modulating the distribution of 14-3-3 adaptor proteins (Eriksson et al., 2009). K17 was also reported to interact with 14-3-3 proteins modulating protein synthesis by interfering with mTOR signaling (Kim et al., 2006). Additionally, mice lacking type II keratins display mislocalization of glucose transporters and downregulation of the protein synthesis machinery (Kellner and Coulombe, 2009; Vijayaraj et al., 2009). Keratin defects exacerbate cell death through increased surface expression of cell death receptors and enhanced activation of apoptotic signaling cascades (Caulin et al., 2000; He et al., 2002; Gilbert et al., 2012). Keratins are also increasingly regarded as stress proteins protecting cells and tissues from stress and injury (Toivola et al., 2010).

In the context of infection, keratins are targeted for degradation during adenovirus and *Chlamydia* infection (Chen et al., 1993; Savijoki et al., 2008), facilitate adhesion of EPEC to HeLa cells (Batchelor et al., 2004), and promote internalization of *Salmonella* (Carlson et al., 2002) and intracellular replication of *Trypanosoma cruzi* (Claser et al., 2008). Interestingly, a recent study showed that in corneal epithelial cells keratin 6a is processed into antimicrobial fragments by the ubiquitin-proteasome system to protect the host against infection (Chan et al., 2018). Despite these observations, the molecular and functional details behind keratin involvement in bacterial pathogenesis remain elusive (Geisler and Leube, 2016) and the possible role of keratins in *L. monocytogenes* infection was never addressed.

*L. monocytogenes* is a facultative intracellular gram-positive pathogen adapted to thrive in diverse environments (Freitag et al., 2009). In humans, it causes listeriosis, a pernicious foodborne disease (Swaminathan and Gerner-Smidt, 2007) that relies on *L. monocytogenes* capacity to enter and survive into epithelial non-phagocytic cells, through the expression of an arsenal of virulence factors (Camejo et al., 2011). *L. monocytogenes* internalization into non-phagocytic cells is mainly driven by the interaction of the bacterial surface proteins InlA and InlB, with their specific host receptors, respectively, E-cadherin and cMet (Mengaud et al., 1996; Shen et al., 2000; Pizarro-Cerdá et al., 2012). The engagement of these host receptors by the bacterial ligands triggers the activation of intracellular signaling pathways that lead to actin polymerization, myosin recruitment and further membrane remodeling, ultimately resulting in the internalization of the bacteria (Ireton et al., 1996, 1999; Bierne et al., 2001; Sousa et al., 2004, 2007; Pizarro-Cerdá et al., 2012; Almeida et al., 2015).

In this study, we assessed the role of epithelial keratins K8 and K18, during *L. monocytogenes* infection. We found that both K8 and K18 are required for successful InlB/cMet-mediated internalization of *L. monocytogenes* and HGF-induced signaling. We also observed that K8 and K18 modulate actin dynamics during InlB-driven internalization. Interestingly, we also showed here that K18, and to a lesser extent K8, control the expression of cMet and other surface receptors such as Transferrin Receptor (TfR) and Integrin β1. Indeed, K18 confers transcript stability, thus regulating post-transcriptionally the expression of such membrane proteins.

## Materials and methods

### Reagents and antibodies

Primary antibodies used are listed in Table 1. Goat anti-mouse HRP or anti-rabbit HRP (P.A.R.I.S.) secondary antibodies were used at 1:2,000 for immunoblotting. For immunofluorescence, secondary antibodies goat anti-rabbit or anti-mouse Alexa Fluor 488 (Invitrogen) and goat anti-mouse or anti-rabbit Cy3 (Jackson Immunoresearch) were used at 1:300. Actin was labeled with Alexa Fluor 647 phalloidin (Invitrogen) or Phalloidin-Tetramethylrhodamine B isothiocyanate (TRITC, Sigma Aldrich). DNA was labeled with 2-(4-Amidinophenyl)-6-indolecarbamidine dihydrochloride (DAPI, Sigma Aldrich). Concanamycin A, MG132 and Actinomycin D were obtained from Sigma Aldrich. HGF was purchased from Peprotech.

**Table 1:** List of antibodies used in this study.

**Antigen**	**Species**	**Applications**	**References**	**Source**
Phosphotyrosine	Mouse	IP (1:360)	4G10, 05-321	Millipore
Actin	Mouse	WB (1:5,000)	A5441	Sigma Aldrich
GAPDH	Mouse	WB (1:15,000)	sc-32233	Santa Cruz Biotechnologies
K8	Mouse	WB (1:450), IF (1:200)	sc-8020	Santa Cruz Biotechnologies
K8	Rabbit	WB (1:10,000), IF (1:400)	ab53280	Abcam
K18	Mouse	WB (1:2,000), IF (1:200)	sc-6259	Santa Cruz Biotechnologies
K18	Rabbit	WB (1:10,000), IF (1:400)	ab52948	Abcam
cMet	Rabbit	WB (1:175), IF (1:150)	Sc-10	Santa Cruz Biotechnologies
TfR	Mouse	WB (1:1500)	13-6800	Thermo
Integrin-β1	Rabbit	WB (1:1,000)	ab52971	Abcam
PI3Kp85	Rabbit	WB (1:1500)	06-195	Millipore
e-cadherin	Rabbit	WB (1:300)	sc-7870	Santa Cruz Biotechnologies
S6	Mouse	WB (1:1,600)	2317	Cell Signaling
Phospho-S6	Rabbit	WB (1:1,000)	4856	Cell Signaling
Akt	Rabbit	WB (1:1,000)	4685	Cell Signaling
P-Akt (S473)	Rabbit	WB (1:1,500)	4060	Cell Signaling

*WB, Western blot; IF, immunofluorescence*.

### Bacterial strains and cell lines

*L. monocytogenes* EGDe strain was grown at 37°C with shaking in brain heart infusion (BHI; BD-Difco). *Listeria innocua* InlB was grown in BHI supplemented with 5 μg/ml erythromycin. *E. coli* K12-*inv* was grown at 37°C with shaking in lysogeny broth (LB) supplemented with 100 μg/ml ampicillin.

HeLa cells (ATCC CCL-2) were cultured in DMEM supplemented with glucose (4.5 g/l), L-glutamine and 10% fetal bovine serum (FBS, Biowest). Caco-2 cells (ATCC HTB-37) were maintained in EMEM supplemented with 20% FBS, L-glutamine, sodium pyruvate and non-essential amino acids. Cells were maintained at 37°C in a 5% CO_2_ atmosphere. Cell culture media and supplements were from Lonza.

### Bacterial infections

Cell infections were performed as described (Reis et al., 2010). For adhesion experiments, bacteria in exponential phase of growth were washed and inoculated at a multiplicity of infection (MOI) of 50. After 30 min, cells were washed five times with phosphate buffered saline (PBS), lysed in 0.2% Triton-X-100 and serial dilutions were plated for quantification of viable bacteria (colony forming units-CFU). For invasion assays, inoculum was prepared as above and cells were infected for 60 min, washed and incubated with medium supplemented with 20 μg/ml gentamicin for 90 min. Cells were washed, lysed with 0.2% Triton-X-100 and serial dilutions plated for CFU counting. For immunofluorescence scoring of adhered and intracellular *L. innocua*-InlB, HeLa cells were inoculated at a MOI of 50 for 30 min, washed and fixed. Before permeabilization, extracellular bacteria were labeled with a rabbit polyclonal antibody raised against *L. innocua* (R6, kindly provided by Prof Pascale Cossart, Institut Pasteur) and an appropriate secondary antibody. Cells were then permeabilized with 0.1% Triton X-100 and total bacteria were labeled with R6 and a secondary antibody coupled to a different fluorochrome. Total and extracellular bacteria were counted under the microscope. For intracellular replication assays, cells were infected with a MOI of 1 for 60 min, washed and incubated with medium complemented with 20 mg/ml gentamicin for 90 min, washed and lysed 2.5, 5, 7, 9, and 12 h after infection. Adhesion and invasion assays were performed in triplicate and repeated at least three times. Replication assays were performed twice in duplicate. For immunofluorescence experiments, cells were infected with *L. innocua* InlB (MOI of 50), washed in PBS and fixed in 3% paraformaldehyde.

### Transfection of siRNA duplexes

HeLa cells were seeded in 24 or 6 well plates and transfected with 46 nM control siRNA-D (sc-44232, Santa Cruz Biotechnology) or with specific siRNAs for K8 or K18 depletion (oligo sequences on Table 2). For partial depletion, we used 13.8 nM of siRNA duplexes. Transfection was performed with HiPerFect (Qiagen) immediately after cell seeding, according to the manufacturer's instructions. Assays were performed 72 h pot-transfection. Transfection of Caco-2 cells was performed with Amaxa Cell line Nucleofector Kit T (Lonza) using program B-024 and following manufacturer's instructions.

**Table 2:** Sequences of siRNA duplexes used in this study.

**siRNA DUPLEXES**		
**Name**	**Oligo Sequence (5′-3′)**	**Source**
K8	Sense: CUGGGAAGGAGGCCGCUAU	SIGMA (Sasi_Hs01_00166576)
	Antisense: AUAGCGGCCUCCUUCCCAG	
K18	Sense: GAGAGGAGCUAGACAAGUA	SIGMA (SASI_Hs01_00145009)
	Antisense: UACUUGUCUAGCUCCUCUCUC	

### Immunoblotting

Protein samples were diluted in Laemmli buffer containing 5% β-mercaptoethanol, resolved on SDS-PAGE gels and transferred to nitrocellulose membranes (Bio-Rad Laboratories). Membranes were blocked in 4% bovine serum albumin (BSA; Sigma Aldrich) or 5% skimmed milk dissolved in TBS-Triton (150 mM NaCl, 20 mM Tris-HCl, pH 7.4, and 0.1% Triton X-100) for 1 h. Primary antibodies were diluted in 2.5% skimmed milk or 4% BSA and incubated overnight at 4°C, incubation with HRP-conjugated secondary antibodies was performed at room temperature for 1 h. ECL (Thermo Scientific) or SuperSignal West Dura Extended Duration Substrate (Pierce) were used for detection of signal on X-ray films (Thermo Scientific) or digitally acquired in a ChemiDoc XRS+ system (Bio-Rad Laboratories).

### Immunoprecipitation assays

Per condition, 2 × 10^6^ cells were washed twice with phosphate-buffered saline (PBS) and serum-starved for 8 h at 37°C and 5% CO_2_. Then, cells were either left untreated or incubated with 150 ng/ml HGF for 5 min. Cells were then washed twice with ice-cold PBS and lysed in 300 μl of lysis buffer [1% NP-40, 50 mM Tris pH 7.5, 150 mM NaCl, 2 mM EDTA, 1 mM AEBSF, PhosSTOP (Roche Pharmaceuticals) and Complete Protease Inhibitor Cocktail (Roche Pharmaceuticals)]. Lysates were centrifuged at 15,000 g for 10 min at 4°C and immunoprecipitated with 0.7 μg of anti-phosphotyrosine antibody (4G10) overnight at 4°C. Immune complexes were captured with 50 μl of PureProteome Protein A magnetic beads (Millipore) at 4°C and washed three times with wash buffer (0.2% NP-40, 50 mM Tris pH 7.5, 150 mM NaCl, 2 mM EDTA, 1 mM AEBSF, PhosSTOP, Complete Protease Inhibitor Cocktail). Immunoprecipitated proteins were eluted and boiled in Laemmli buffer.

### Cell surface biotinylation assay

Cell surface protein biotinlyation was performed using the EZ-Link Sulfo-NHS-Biotinylation kit (Thermo Scientific) as described in Martins et al. (2012) and accordingly to manufacturer's protocol. In brief, 2 × 10^6^ cells were washed with ice cold PBS (pH 8), incubated with 2 mM Sulfo-NHS-biotin (2 h at 4°C), washed with cold 100 mM glycine in PBS (pH 7.2), harvested, and lysed in RIPA (sc-364162, Santa Cruz Biotechnology). Cell extracts (90 μg) were incubated with 50 μl of neutravidin agarose resin (Thermo Scientific) overnight at 4°C, with rotation. Resin was washed and captured biotinylated proteins were eluted with Laemmli buffer.

### Immunofluorescence microscopy

Cells were fixed in 3% paraformaldehyde (10 min), quenched with 20 mM NH_4_Cl (1 h), permeabilized with 0.2% Triton X-100 (6 min), washed and blocked with 1% BSA in PBS. Antibodies were diluted in the blocking buffer. Coverslips were incubated with primary antibodies (1 h), washed in PBS, incubated with secondary antibodies, phalloidin TRITC or Alexa 647 and DAPI for 45 min, and mounted onto microscope slides with Aqua-Poly/Mount. Images were analyzed and collected with an epifluorescent Zeiss Axio Imager Z1 microscope or an Olympus BX63 microscope. When necessary, Z-stacks were deconvoluted with Huygens Professional Software (SVI, Netherlands) and projected with ImageJ software (NIH).

### Ruffle formation assays

Cells were serum starved for 7 h, stimulated with 150 ng/ml HGF for 5 and 10 min, fixed in 3% paraformaldehyde (PFA) and processed for immunofluorescence. Cells with at least one actin rich membrane ruffle were scored as ruffle-positive, cells with no ruffles were considered ruffle-negative. Data were obtained from four independent experiments, for which at least 180 cells/condition were analyzed.

### Rates of total protein synthesis

Cells (2 × 10^6^) were labeled with ^35^S-methionine (22.5 uCi/ml, PerkinElmer) in methionine free DMEM (2 h at 37°C), washed twice with PBS and lysed in RIPA buffer. Protein samples diluted in Laemmli buffer were loaded into a 10% polyacrylamide gel and resolved by SDS-PAGE, followed by autoradiography.

### Quantitative real-time PCR

Total RNAs were isolated using TripleXtractor (GRiSP), following manufacturer's protocol. Purified RNAs (1 μg) were reverse transcribed with iScript cDNA Synthesis Kit (Bio-Rad Laboratories). Quantitative real-time PCR (qRT-PCR) was performed in 10 μl reactions containing 5 μl iTaq Universal SYBR Green Supermix (Bio-Rad Laboratories), 1 μl of cDNA and 0.1 μl of 10 μM forward and reverse primers (Table 3), using the following protocol: 3 min (95°C), followed by 40 cycles of 10 s (95°C), 20 s (55.6°C), and 20 s (72°C). Each target gene was analyzed in triplicate and blank control was included for each primer pair. The comparative threshold method (ΔΔCt) was used to analyze the amplification data after normalization of the test and control sample expression values to a housekeeping reference gene (GAPDH).

**Table 3:** Sequences of primers used in this study.

**PRIMER SEQUENCES (5′-3′)**	
cMet	Fw: CCCTATCAAATATGTCAACG
	Rev: TCAGAAGTGTCCTATTAAAGC
TFRC	Fw: GGAATATGGAAGGAGACT
	Rev: ATAGTGATCTGGTTCTACA
ITGB1	Fw: GCCATTATTATGATTATCCTTCT
	Rev: GTTCCTACTGCTGACTTAG
GAPDH	Fw: CCTCAAGATCATCAGCAATG
	Rev: CACGATACCAAAGTTGTCAT

### mRNA stability assays

Cells were incubated with Actinomycin D (5 μg/ml) for 1 and 2 h to inhibit *de novo* RNA synthesis. Cells were harvested and RNAs isolated, reverse transcribed and analyzed by qRT-PCR. GAPDH was used as reference gene and fold changes were normalized to the untreated control. At least three independent experiments were performed for each gene of interest.

### InlB-coated beads assays

Purified InlB (350 μg) was covalently coupled to 200 μl of a 4% aqueous suspension of 1.0 μm carboxylated modified latex beads (Thermo Scientific), following manufacturer's instructions. To synchronize the uptake, HeLa cells were incubated with InlB-coated beads at 4°C, centrifuged (5 min at 320 g) and incubated at 37°C. Cells were washed in ice cold PBS and processed for immunofluorescence. At least 20 cells and more than 150 beads were analyzed per condition, in at least three independent experiments. To assess internalization, extracellular beads were stained with anti-InlB B4-6 antibody (Braun et al., 1999) before cell permeabilization. Samples were then analyzed in a high-throughput widefield fluorescence microscope (IN Cell Analyzer 2000, GE Healthcare). Total beads number was quantified in brightfield. Per condition, at least 500 cells and 5,000 beads were analyzed.

### Statistical analyses

Statistical analyses were performed with Prism 7 software (GraphPad) using: two-tailed unpaired Student's *t*-test for comparison of means between two samples, one-tailed *t-*test for comparisons with samples arbitrarily fixed to 100 and one-way ANOVA with Dunnett's *post-hoc* analysis to compare different means in relation to a control sample. Differences were not considered statistically significant for *p* ≥ 0.05

## Results

### K8 and K18 favor InlB/cMet-mediated *L. monocytogenes* cellular invasion

**Figure 1:**
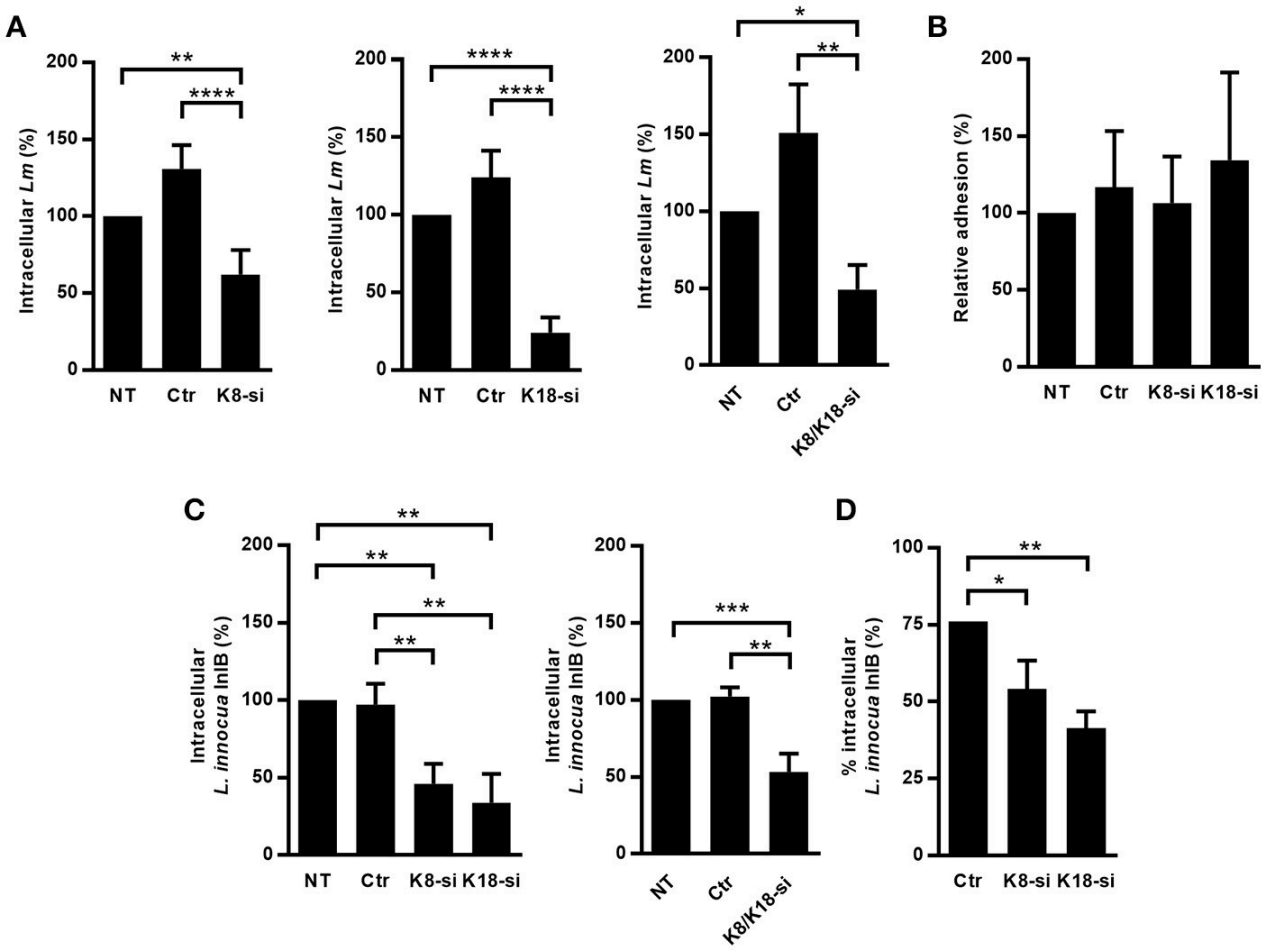
K8 and K18 promote *Listeria* infection of HeLa cells. **(A)** Intracellular levels of *L. monocytogenes* were determined by gentamicin protection assay and CFU counting in HeLa cells left untransfected (NT) or transfected with either control (Ctr) or siRNA specifically targeting K8 (K8-si, left panel), K18 (K18-si, middle panel) and both (K8/K18-si, right panel). **(B)** Adhesion of *L. monocytogenes* was assessed in HeLa cells left unstransfected (NT) or transfected with Ctr, K8 or K18 siRNA. **(C,D)** Intracellular levels of *L. innocua* expressing InlB (*L. innocua* InlB) were determined **(C)** by gentamicin protection assays and CFU counting in HeLa cells left unstransfected (NT) or transfected with Ctr or specific siRNA targeting K8 (K8-si left panel), K18 (K18-si, left panel) and both (K8/K18-si, right panel) or by **(D)** immunofluorescence scoring of extracellular and total bacteria. Values of intracellular or adherent bacteria in NT cells were normalized to 100% and the levels of infection in the remaining conditions are expressed as relative values. Values represent the mean ± S.E. of at least three independent experiments, each done in triplicate. Statistically significant differences are indicated: ^*^p < 0.05, ^**^p < 0.01, ^***^p < 0.001, and ^****^p < 0.0001.

We assessed the relevance of keratins during *L. monocytogenes* cellular infection of epithelial cell lines, which mainly express K8 and K18 (Moll et al., 2008). HeLa and Caco-2 cells were depleted for K8 and/or K18 through an siRNA approach and intracellular *L. monocytogenes* numbers were evaluated by gentamicin protection assays (Almeida et al., 2015). Numbers of intracellular bacteria were significantly decreased in K8, K18, and K8/K18-depleted HeLa cells, as compared to control cells (Figure 1A). In turn, in Caco-2 cells, the depletion of K8 and/or K18 had no effect on the number of intracellular bacteria (Supplemental Figure 1). Furthermore, K8 and/or K18 depletion in HeLa had no impact on the ability of bacteria to adhere to the cells (Figure 1B). The efficiency of K8 and/or K18 depletion in the different cell lines was confirmed by western blot analysis, using GAPDH as loading control (Supplemental Figure 2). Altogether these data indicate that K8 and K18 are required for internalization of *L. monocytogenes* in HeLa cells, but not in Caco-2 cells.

*L. monocytogenes* invasion of epithelial cells is mainly driven by the interaction of the bacterial surface proteins InlA and InlB with their host receptors E-cadherin and cMet, respectively (Mengaud et al., 1996; Shen et al., 2000). In HeLa cells *Listeria* internalization largely occurs through the InlB/cMet axis, while in Caco-2 cells invasion relies essentially on the InlA/E-cadherin interplay (Shen et al., 2000; Sousa et al., 2007). The observation that keratins are specifically required for *L. monocytogenes* infection of HeLa, but not Caco-2 cells suggested that K8 and K18 are particularly important for the InlB/cMet-mediated internalization pathway. To confirm this, we evaluated in K8- and/or K18-depleted HeLa cells the internalization of *L. innocua* expressing InlB (*L. innocua*-InlB), which invades non-phagocytic cells exclusively through the InlB pathway (Braun et al., 1999). Similarly to what we observed for *L. monocytogenes*, internalization of *L. innocua*-InlB was compromised in K8- and/or K18-depleted cells (Figures 1C,D), thus confirming that K8 and K18 are required for efficient InlB/cMet-mediated entry of *L. monocytogenes* into human epithelial cells. Finally, we found that K8 and K18 are not involved in intracellular replication of *L. monocytogenes* in HeLa cells (Supplemental Figure 3). Taken together, these results demonstrate that K8 and K18 play a key role in InlB/cMet-mediated internalization of *L. monocytogenes*.

### K8 and K18 accumulate at InlB-mediated internalization sites

**Figure 2:**
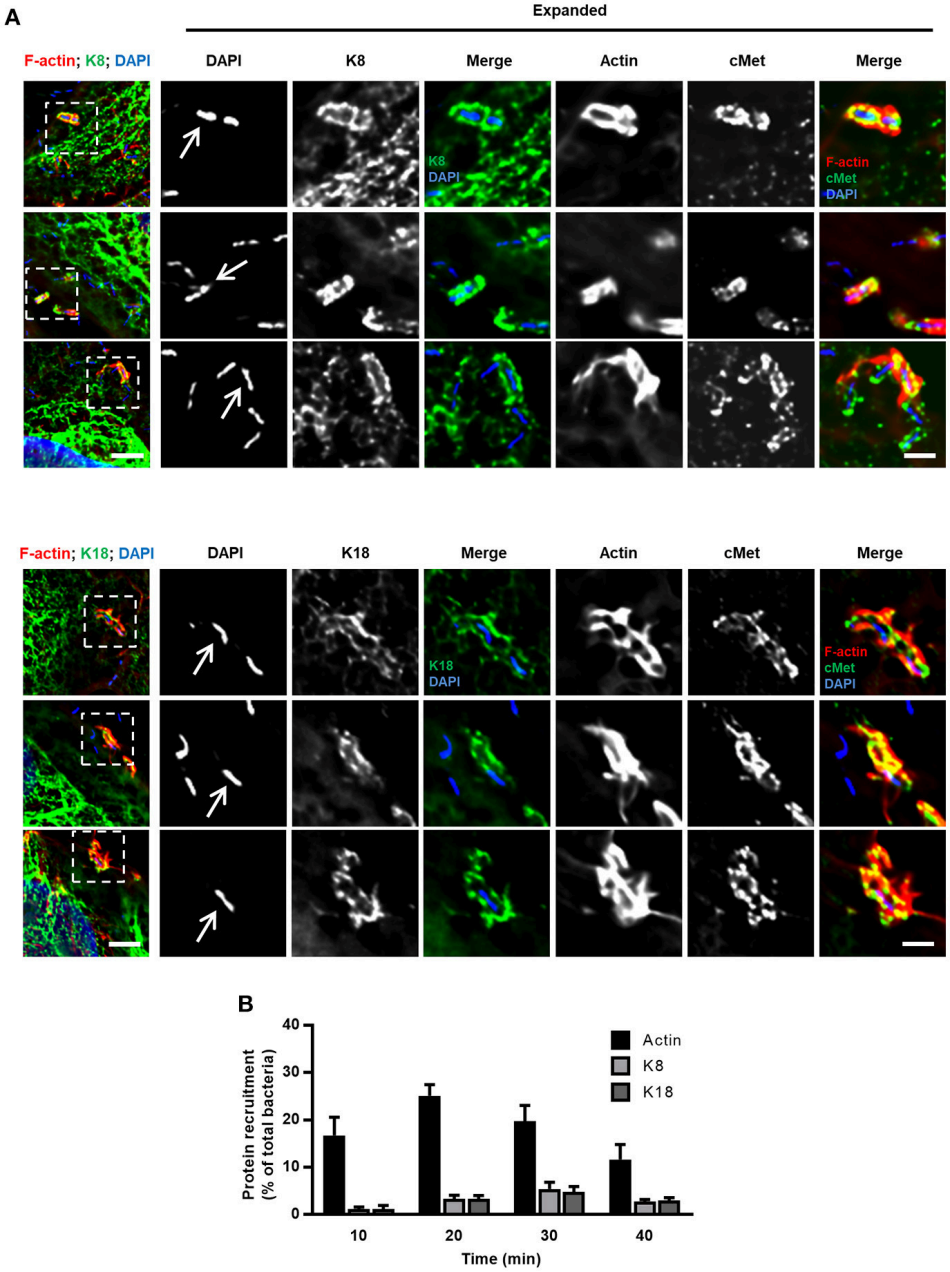
K8 and K18 are recruited at the bacterial entry site during InlB-mediated cellular invasion. **(A)** Representative widefield microscopy stack projections of HeLa cells incubated with *L. innocua* InlB for 5 min, fixed and immunostained for cMet (green) and for K8 (upper panels, green) or K18 (lower panels, green). F-actin was stained with phalloidin (red), DNA with DAPI (blue). Scale bar, 5 μm. Arrows indicate bacteria that display accumulation of K8, K18, cMet, and F-actin at their vicinity. Insets show high-magnification images. Scale bar, 2 μm. **(B)** Quantification of K8, K18, and actin recruitments to the entry site of *L. innocua* InlB. Results are expressed as the percentage of total number of bacteria associated to cells. Values are the mean ± S.E. of at least three independent experiments.

To further characterize the role of K8 and K18 in InlB-driven invasion of *Listeria*, we investigated their cellular distribution in infected cells. HeLa cells were infected with *L. innocua-*InlB, fixed and processed for immunofluorescence. K8, K18, and cMet were immunolabeled using specific antibodies, DNA was stained using DAPI and actin was detected by phalloidin staining. K8 and K18 accumulated at the vicinity of the bacteria within minutes after infection (Figure 2A), together with F-actin and cMet, two proteins already described to accumulate at sites of entering bacteria (Bierne et al., 2001). Quantifications of actin, K8 and K18 recruitments to the bacterial entry site were performed at different time points and are shown in Figure 2B. Although K8 and K18 recruitments were less frequent than actin recruitments, these observations further support the involvement of K8 and K18 in early steps of *Listeria* cellular invasion.

### K8 and K18 modulate actin dynamics at InlB-mediated entry sites

**Figure 3:**
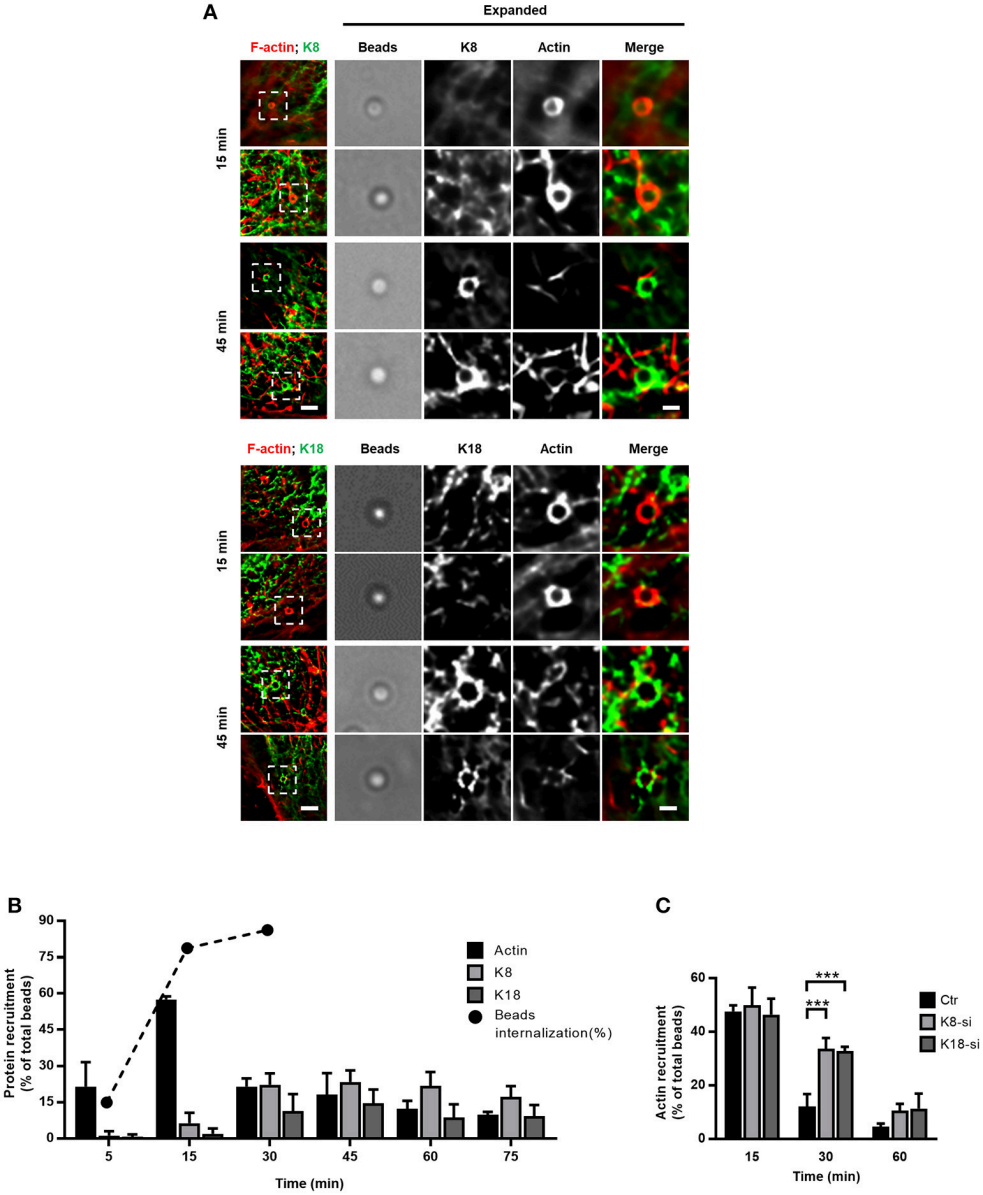
K8 and K18 assist actin depolymerization during later stages of internalization. **(A,B)** Kinetic analysis of actin, K8 and K18 recruitments during internalization of InlB-coated latex beads. **(A)** Stack projections of widefield microscopy images of HeLa cells incubated with InlB-coated latex beads for different periods of time, fixed, immunostained for K8 or K18 (green) and labeled for F-actin with TRITC-phalloidin (red). Scale bar, 3 μm. Insets show high-magnification images. Scale bar, 1 μm. **(B)** Quantification of beads positive for K8, K18, or actin recruitment. Results are expressed as the percentage of particles associated with either protein in relation to the total number of particles associated to cells. The total number of beads was determined in brightfield. Values are the mean ± S.E. of at least three independent experiments. For determination of beads internalization, extracellular beads were stained with anti-InlB before cell permeabilization and total beads number quantified in brightfield. Values are shown in percentage and are representative of two independent experiments. **(C)** Quantification of InlB-coated latex beads associated to polymerized actin in HeLa cells transfected with control (Ctr) or specific siRNA targeting K8 (K8-si) or K18 (K18-si). Cells were incubated with InlB-coated latex beads for 15, 30, and 60 min, fixed and stained for F-actin. Beads displaying actin recruitment were considered recruitment-positive. The total number of beads associated to cells was determined in brightfield. Values represent the mean ± S.E. of at least three independent experiments. Statistically significant differences are indicated: ^***^p < 0.001.

The entry process of *L. monocytogenes* into epithelial cells is a dynamic process that engages actin rearrangements and membrane remodeling (Pizarro-Cerdá et al., 2012). To gain better understanding of the dynamics of keratin recruitment to the sites of internalization and to further dissect the role of keratins in such process, we used InlB-coated beads whose entry mimics the InlB/cMet-mediated *L. monocytogenes* internalization (Braun et al., 1999; Pizarro-Cerdá et al., 2002). HeLa cells were incubated with InlB-coated beads for different periods of time and processed for immunofluorescence analysis. As we reported for *L. innocua-*InlB (Figure 2), K8 and K18 accumulated around entering InlB-coated beads (Figure 3A). We quantified the percentage of InlB-coated beads associated with actin, and K8 and K18 recruitments at different incubation time points (Figure 3B). As previously reported (Bierne et al., 2001), actin filaments rapidly accumulate at the vicinity of InlB-coated beads. Actin recruitment peaked at 15 min, with 60% of the beads associated to actin filaments, and promptly decreased afterwards. In turn, K8 and K18 recruitments to the vicinity of InlB-coated beads appeared later, being maximum at 30 min and sustained for longer incubation periods (Figure 3B). These data indicate that actin and keratin recruitments are sequential events during the internalization process of beads. To assess the potential role of K8/K18 on actin dynamics, HeLa cells depleted for K8 or K18 were incubated with InlB-coated beads for different periods of time, processed for immunofluorescence and actin recruitments around beads were quantified. In accordance to our results in Figure 3B, in control cells actin rings surrounding InlB-coated beads peaked at 15 min after incubation to then rapidly decrease at later time points (Figure 3C). In K8- and K18-depleted cells, while the percentage of InlB-coated beads associated to actin rings were equivalent to those of control cells at 15 min, they remain significantly higher at 30 min (Figure 3C). In cells partially depleted for K8 or K18 the levels of InlB-beads associated to actin rings are intermediate between those of control and more robustly depleted cells (Supplemental Figure 4). Thus, the persistence of polymerized actin around entering InlB-beads depends on the expression levels of K8 and K18. Low K8 and K18 expression increases the time during which polymerized actin associates with InlB-entering beads. These data strongly suggest a role for K8/K18 in the regulation of actin depolymerization necessary for the effective internalization of particles (Bierne et al., 2001).

### K8 and K18 control HGF/cMet-mediated signaling

**Figure 4:**
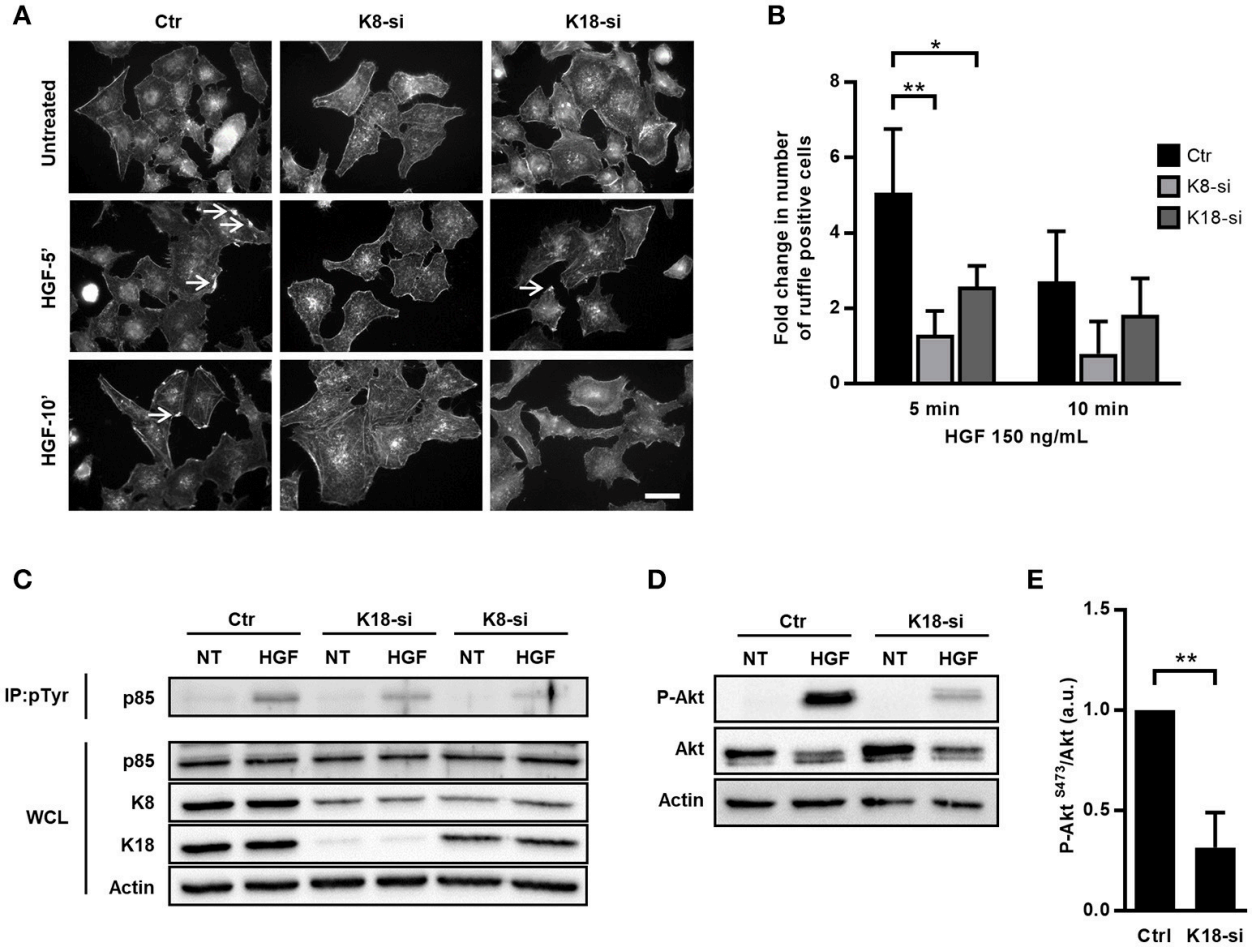
K8 and K18 mediate cMet downstream signaling. **(A)** Immunofluorescence microscopy images of control (Ctr), K8 (K8-si), or K18 (K18-si) depleted HeLa cells left untreated or incubated with HGF (150 ng/ml) for 5 and 10 min (HGF-5′ and HGF-10′). Cells were fixed and stained for actin with TRITC-phalloidin. Images show the actin-rich membrane ruffles (arrows) induced by the HGF stimulation of cMet. Scale bar, 20 μm. **(B)** Quantification of actin-rich membrane ruffles in Ctr, K8- and K18-depleted cells. Cells without ruffles were considered ruffle-negative, whereas cells with at least one actin-rich membrane ruffle were scored as ruffle-positive. Values result from four independent experiments and are expressed as fold change with respect to untreated control cells. **(C)** Ctr, K8 and K18-depleted HeLa cells were incubated with 150 ng/ml HGF for 5 min, washed and lysed. Tyrosine phosphorylated proteins were immunoprecipitated (IP: pTyr) from whole cell lysates (WCL) and p85 was detected by immunoblot (p85) in IP fractions and WCL. Detection of actin was used as loading control. **(D)** Immunoblot to detect P-Akt (S473), total Akt and actin on total extracts of Ctr and K18-depleted HeLa cells left untreated (NT) or incubated with 150 ng/ml HGF for 5 min. **(E)** Densitometry analysis of the ratio of P-Akt (S473) over total Akt, in conditions of HGF stimulation. For control cells the value was arbitrarily fixed to 1. Values represent the mean ±S.E. of three independent experiments. Statistically significant differences are indicated: ^*^p < 0.05 and ^**^p < 0.01.

The data obtained in the context of *Listeria* InlB/cMet-mediated internalization suggested a role for K8/K18 in cMet downstream signaling. It was previously demonstrated that InlB triggers cMet similarly to its natural ligand, the hepatocyte growth factor (HGF) (Li et al., 2005). Indeed, both HGF and InlB bind and activate cMet, and share common downstream signaling cascades that trigger MAPK and PI3-kinase pathways to promote either cell migration and proliferation or bacterial internalization (Ireton et al., 1996; Tang et al., 1998; Shen et al., 2000; Copp et al., 2003). To assess the potential role of K8/K18 in the HGF/cMet signaling pathway, we analyzed and quantified the formation of HGF-induced membrane ruffles in control, K8- and K18-depleted cells. Cells were stimulated with HGF for different time periods, fixed and processed for immunofluorescence. Membrane ruffles were detected through actin staining, which locally accumulate at the cortex of the cells undergoing ruffling (Figure 4A). Cells with at least one actin-rich membrane ruffle were scored as positive. While in control cells, HGF stimulation quickly induced the formation of actin rich ruffles that peaked at 5 min, in K8-and K18-depleted cells ruffle formation was compromised even at longer time points (Figure 4B). These data indicate that K8 and K18 also play a role in HGF-induced cMet signaling.

To further dissect the role of K8/K18 in cMet downstream signaling, we assessed HGF-dependent activation of PI3-kinase (PI3K) in control, K8 and K18-depleted cells. Serum-starved cells were incubated with HGF for 5 min, washed and lysed. Cell lysates were subjected to anti-phosphotyrosine immunoprecipitation and revealed for the PI3K p85 subunit. Western blots of phosphotyrosine enriched protein fractions showed decreased levels of the PI3K p85 subunit in K8/K18-depleted cells (Figure 4C), indicating an impaired association of PI3K with tyrosine phosphorylated proteins in absence of keratins and suggesting a defect in PI3K activation. In addition, K18-depleted cell lysates were directly subjected to immunoblot analysis to detect phosphorylation of Akt on serine 473 (P-Akt, S473), a direct downstream target of PI3K activity (Basar et al., 2005; Vanhaesebroeck et al., 2012; Gessain et al., 2015). As expected, in control cells HGF stimulation induced robust phosphorylation of Akt, which is extensively compromised in K18-depleted cells (Figures 4D,E). Together, these results demonstrate that K18, and to a lesser extent K8, are important players in the cMet-mediated signaling cascade and suggest that K8/K18 are involved upstream the activation of PI3K.

### cMet expression is dependent on K8 and K18

**Figure 5:**
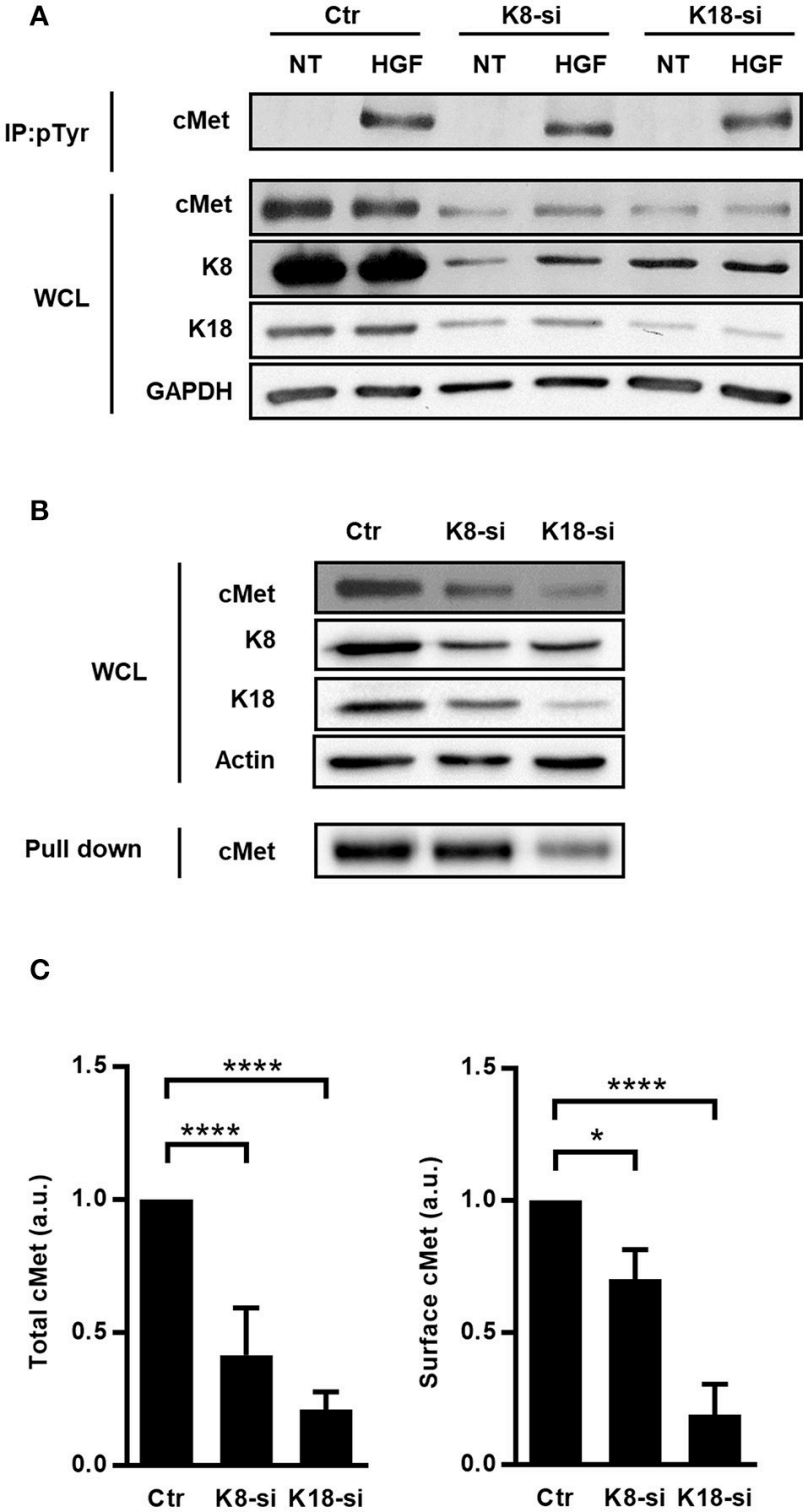
Total expression, surface localization and activation of cMet are perturbed in cells expressing low levels of K8 and K18. **(A)** HeLa cells transfected with Ctr, K8, and K18-targeting siRNAs were left untreated (NT) or incubated with 150 ng/ml HGF for 5 min, washed and lysed. Tyrosine phosphorylated proteins were immunoprecipitated (IP: pTyr) from whole cell lysates (WCL) and cMet was analyzed by immunoblot (cMet) in IP fractions and WCL. GAPDH detection was used as loading control. **(B)** Surface exposed proteins of control (Ctr), K8- (K8-si), and K18-depleted (K18-si) HeLa cells were biotinylated and recovered from total cell extracts following neutravidin pull down assays. Biotinylated samples, corresponding to surface exposed proteins, and whole cell lysates (WCL) were immunoblotted to detect cMet, K8, K18 and actin. **(C)** Quantifications of cMet in WCL (left panel) and in biotinylated samples (right panel) from at least three independent experiments. Statistically significant differences are indicated: ^*^p < 0.05 and ^****^p < 0.0001 (a.u., arbitrary units).

To identify the precise role of K8/K18 in cMet-mediated signaling upstream PI3K activation, we assessed the expression and activation levels of cMet. Indeed, both InlB-mediated *L. monocytogenes* internalization and the formation of HGF-triggered membrane ruffles rely on the surface expression and auto-phosphorylation of cMet on tyrosine residues (Shen et al., 2000). Interestingly, K8 and K18 were reported as modulators of the expression and/or localization of surface proteins such as the apoptotic receptor Fas, the chloride transporter DRA and the cystic fibrosis transmembrane conductance regulator (CFTR) (Gilbert et al., 2001; Duan et al., 2012; Asghar et al., 2016). Thus, this raises the possibility that keratins may also modulate cMet expression and/or activity. We evaluated the levels of total cMet expression and activation upon HGF stimulation in whole cell lysates of control, K8- and K18-depleted cells. Surprisingly, we observed that cells depleted for K8 or K18 displayed reduced levels of total cMet (Figures 5A–C). Nevertheless, upon HGF stimulation cMet activation, as measured by phosphotyrosine immunoprecipitation assays, was detected at variable extents in those cells (Figure 5A). To determine if the low levels of total cMet expression observed in K8- and K18-depleted cells also result in a reduction of cell surface associated cMet, we specifically analyzed and quantified cell surface expression of cMet by performing biotinylation assays. Surface proteins of control, K8- and K18-depleted cells were labeled using a membrane-impermeable biotinylation reagent, recovered with neutravidin-coupled beads and analyzed by immunoblot. In agreement with the observed reduced levels of total cMet expression, K8 or K18 depletion resulted in decreased levels of cMet at the cell surface (Figures 5B,C). Altogether, these data clearly indicate that K8 and K18 control the global and surface expression of cMet, thus impacting cMet-mediated signaling events elicited by ligands such as HGF and *L. monocytogenes* InlB.

### K18 controls the expression of other transmembrane receptors

**Figure 6:**
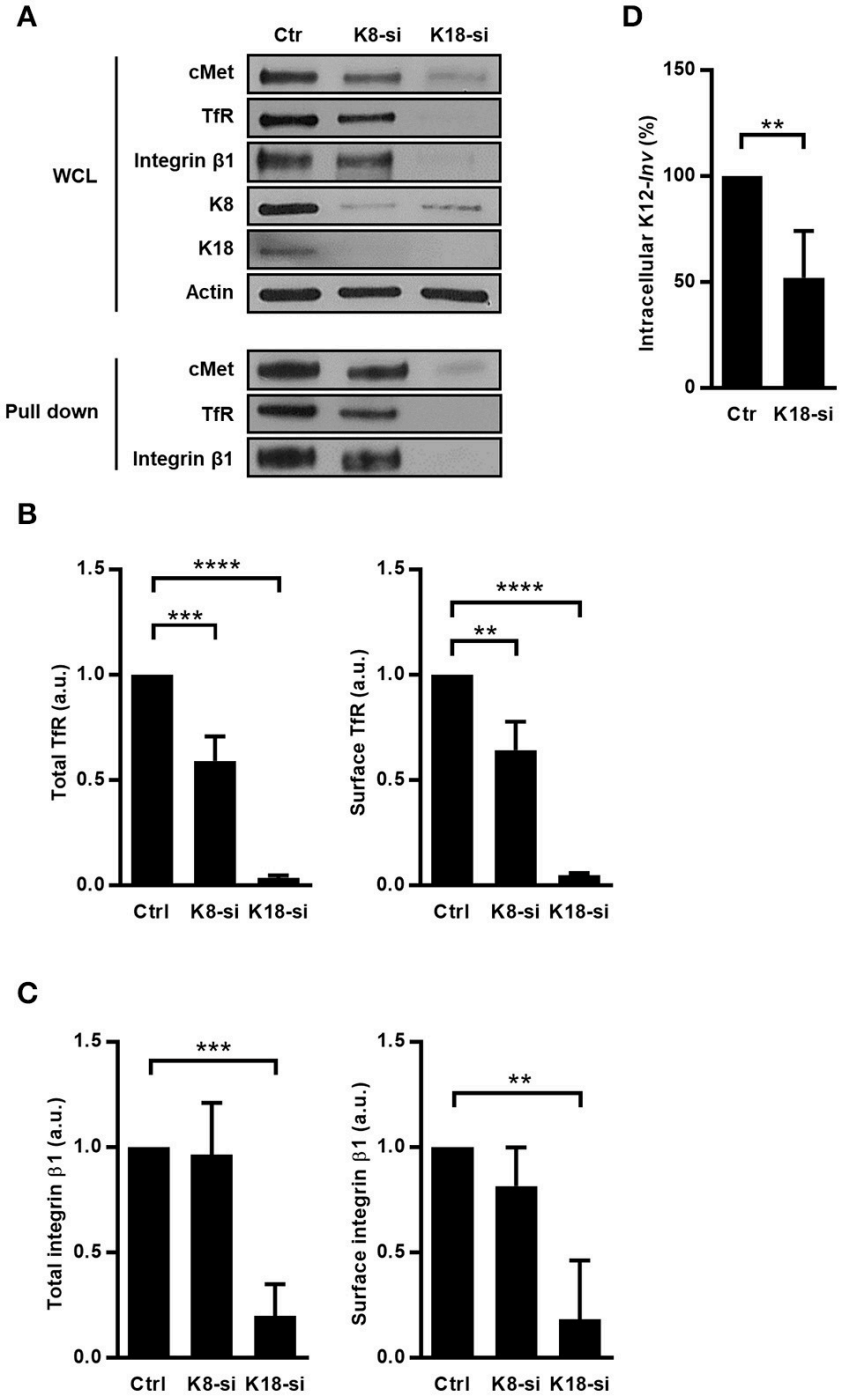
K8 and K18 depletion perturbs expression and surface localization of transmembrane receptors. **(A)** Surface proteins of control (Ctr), K8- (K8-si), and K18-depleted (K18-si) HeLa cells were biotinylated, recovered from total cell extracts and pulled down using neutravidin beads. Biotinylated samples, which corresponds to surface exposed proteins, and whole cell lysates (WCL) were immunoblotted to detect cMet, TfR, and integrin β1, together with Actin, K8, and K18. **(B)** Quantifications of TfR in WCL (left panel) and in biotinylated samples (right panel) from at least three independent experiments. **(C)** Quantifications of integrin β1 in WCL (left panel) and in biotinylated samples (right panel) from at least three independent experiments (a.u., arbitrary units). **(D)** Functional impact of K18 in the expression of ITGB1 was assessed by gentamicin survival assay and CFU counting in K18-depleted HeLa cells (K18-si) incubated with invasive *E. coli* K12 expressing the *Y. pseudotuberculosis* invasin (K12-inv). Values of intracellular bacteria in Ctr cells were normalized to 100% and the entry levels in K18-si cells are expressed as relative values. Values are the mean ± S.E. of three independent experiments, each done in triplicate. Statistically significant differences are indicated: ^**^p < 0.01, ^***^p < 0.001, and ^****^p < 0.0001.

Given that K8 and K18 were already reported as modulators of expression of surface proteins (Duan et al., 2012; Asghar et al., 2016) and taking into account our data, we hypothesized that K8 and K18 may have a broad role in controlling the expression of surface receptors. To investigate this hypothesis, we assessed the impact of K8 and K18 on the expression and surface localization of transferrin receptor (TfR) and integrin β1 in HeLa cells. Immunoblot analysis of whole cell lysates and surface biotinylated fractions revealed that K18 depletion resulted in a striking decrease of total and cell surface associated levels of both TfR and integrin β1 (Figures 6A–C). K8 depletion lead to a mild reduction of total and surface localized TfR and had no significant effect on the expression of integrin β1 (Figures 6A–C). Additionally, we performed similar experiments in Caco-2 cells and observed that K18 depletion also lead to a reduction of total and surface levels of cMet, TfR, and integrin β1 (Supplemental Figure 5), suggesting that the mechanism through which K18 regulates the expression of these proteins is conserved in different cellular systems. Interestingly, the expression of E-cadherin is not dependent on keratins (Supplemental Figure 4).

To functionally assess the impact of integrin β1 downregulation induced by K18 depletion, we measured levels of internalization of *E. coli* K12 expressing the *Yersinia* invasin (K12-*inv*), which is strictly dependent on the interaction of the bacterial invasin with the host integrin β1 (Isberg and Leong, 1990). As expected, K18-depleted cells showed reduced levels of intracellular K12-*inv* (Figure 6D). Taken together, these results demonstrate that K18, and to a lesser extend K8, control the expression of some cell surface receptors, in turn modulating signaling events taking place downstream the engagement of these receptors.

### Protein synthesis and stability do not depend on K18 expression

**Figure 7:**
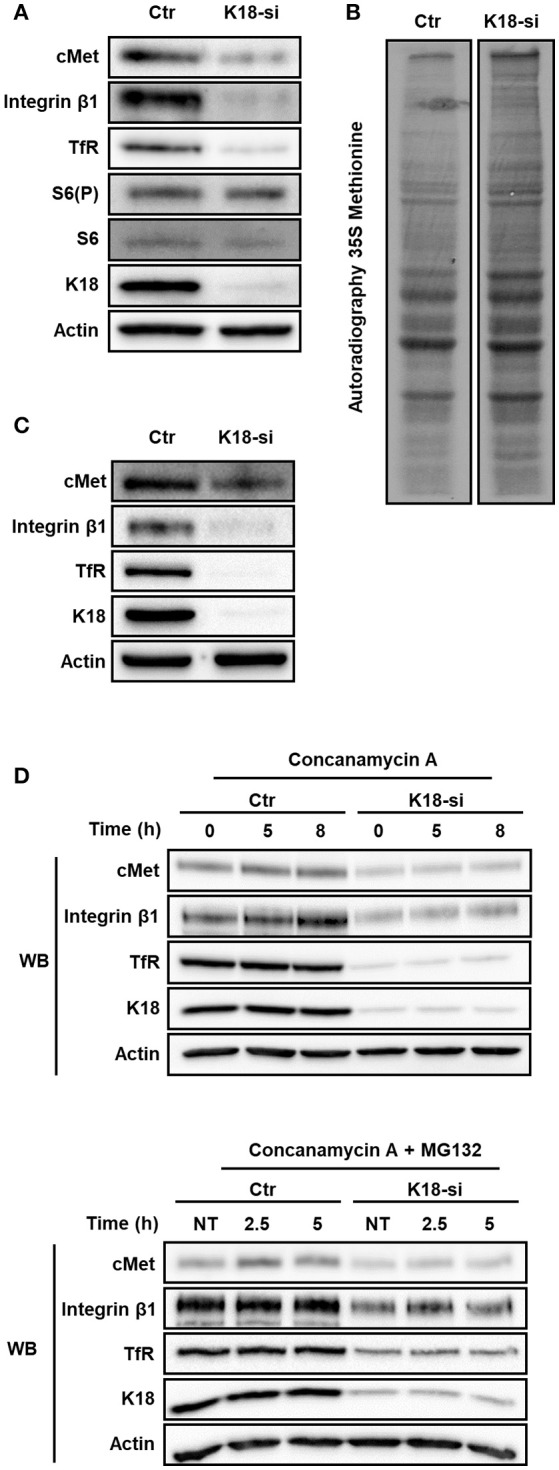
K18 depletion does not dampen mTOR/S6K signaling, global protein translation and receptor degradation. **(A)** Activation of mTOR/S6K signaling pathway in K18 (K18-si) depleted HeLa cells was assessed by immunoblotting whole cell extracts against phosphorylated S6 (S6(P)), total S6, cMet, K18, and Actin as loading control. Immunoblot representative of three different experiments. **(B)** Rate of total protein synthesis was assessed by ^35^S-methionine incorporation of HeLa cells transfected with control (Ctr) or K18 targeting (K18-si) siRNA. Autoradiography representative of two independent experiments. **(C)** Depletion efficiency of the samples that were used for the ^35^S-methionine incorporation assay. **(D)** After transfection with control (Ctr) or siRNA targeting K18 (K18-si), HeLa cells were incubated with 100 nM of the lysosomal inhibitor Concanamycin A alone (*upper* panel) or together with the proteasomal inhibitor 10 μM MG132 (*lower* panel) for different periods of time. Lysates were collected and immunoblotted for cMet, TfR, integrin β1, K18, and Actin as a loading control. Immunoblots are representative of at least two independent experiments.

The decrease of total levels of cMet, TfR, and integrin β1 observed in K18-depleted cells lead us to put forward the possibility that protein synthesis would be impaired in these cells. Indeed, K8/18 depletion was reported to lead to reduced protein synthesis in human H4 neuroglioma cells (Galarneau et al., 2007). In addition, mTOR signaling and, consequently, protein synthesis were shown to be impaired in keratinocytes lacking Keratin 17 (Kim et al., 2006). We thus assessed if mTOR signaling and global protein synthesis were compromised in K18-depleted HeLa cells, which would account for the reduced levels of cMet, TfR, and integrin β1. The ribosomal protein S6 is the target of p70S6K, a major mTOR effector (Magnuson et al., 2012), and S6 phosphorylation is thus used as a readout for mTOR activity (Biever et al., 2015; González et al., 2015). To evaluate the involvement of K18 in mTOR signaling activity, we thus analyzed the level of phosphorylated S6 in control and K18-depleted HeLa cells. S6 phosphorylation was detected in both control and K18-depleted cells (Figure 7A), indicating that mTOR activity is not compromised and suggesting that mTOR-dependent protein synthesis is not impaired in absence of K18. To assess the rate of bulk protein synthesis, control or K18-depleted cells were incubated with radiolabeled methionine to be incorporated into newly synthesized proteins. Total protein extracts were resolved by SDS-PAGE and labeled proteins detected by autoradiography. No major defect was detected in K18-depleted as compared to control cells (Figure 7B), indicating that the global initiation rate of translation is not compromised in cells lacking K18. The same samples were used in immunoblot to confirm the down-regulation of cMet, integrin β1, and TfR expression in K18-depleted cells (Figure 7C). These observations demonstrate that K18 does not impact significantly protein translation and *de novo* synthesis and suggest that other mechanisms should govern the K18-dependent expression of cMet, TfR and integrin β1.

Interestingly, K18 was previously reported to enhance the stability of the surface protein CFTR (Duan et al., 2012). We thus hypothesized that K18 could promote the stability of cMet, integrin β1, and TfR by minimizing their degradation. To investigate this hypothesis, control and K18-depleted HeLa cells were treated with the lysosomal inhibitor concanamycin A alone or together with the proteosomal inhibitor MG132 for different time periods. Cell extracts were immunoblotted for cMet and TfR. In both conditions tested, control and K18-depleted cells behaved similarly and no significant accumulation of cMet, integrin β1, and TfR was detected upon blockage of protein degradation (Figure 7D).

Altogether, these results indicate that the downregulation in the expression of cMet, TfR, and integrin β1 detected in K18-depleted cells is not due to a defect on protein synthesis or stability.

### K18 promotes transcripts stability

**Figure 8:**
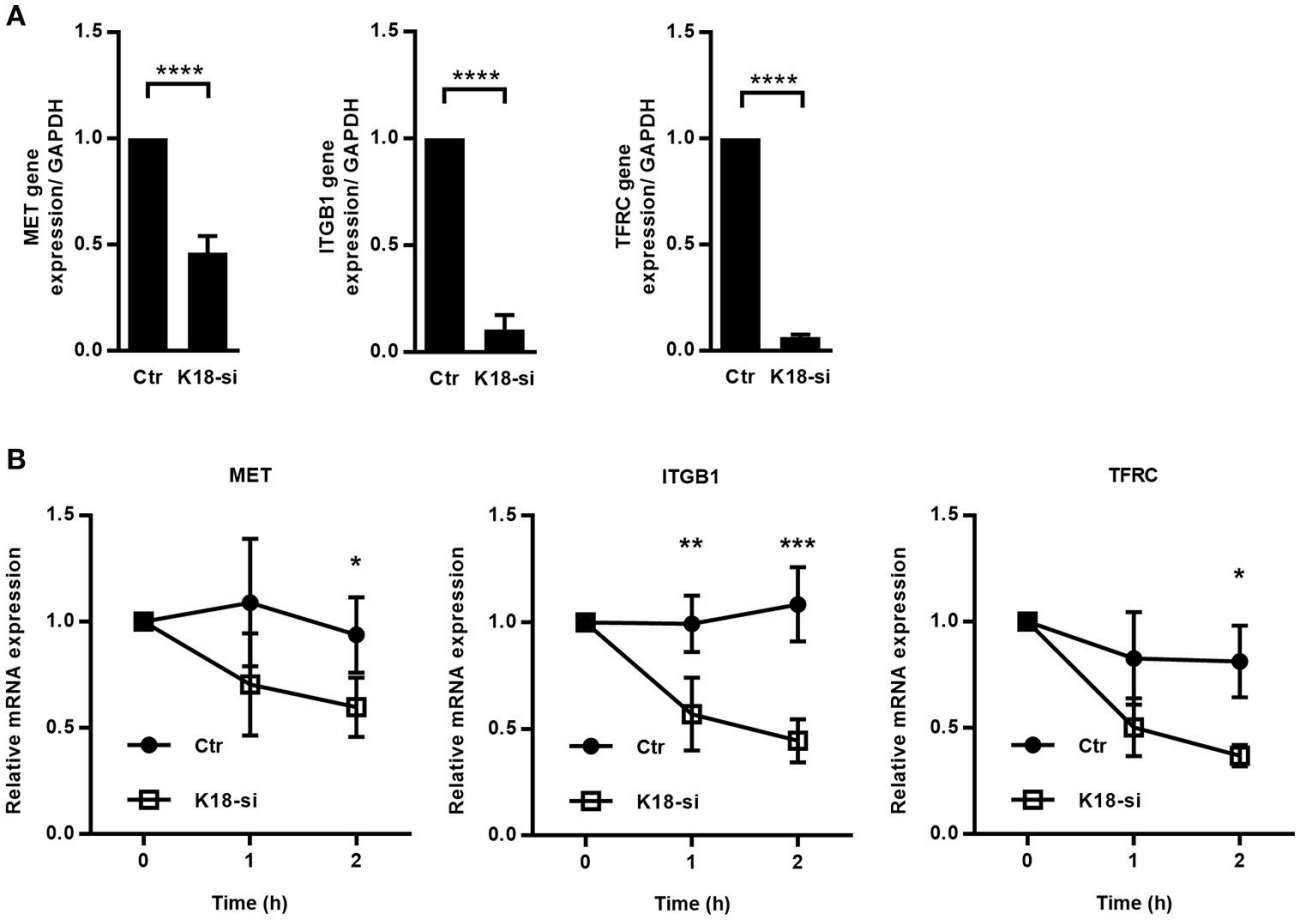
K18 favors expression of cMet, TfR and integrin β1, by promoting transcript stability. **(A)** mRNAs were extracted from control (Ctr) and K18-depleted (K18-si) HeLa cells and qRT-PCR was performed using *GAPDH* as a housekeeping gene. Data are represented as mean ± S.E. from at least three independent experiments **(B)** Control and K18 depleted cells were left untreated or were treated with 5 μg/ml of the transcriptional inhibitor Actinomycin D for different periods of time. Transcript levels for cMet, TfR, and integrin β1 were determined by qRT-PCR. Fold changes are relative to *GAPDH* and were normalized to untreated control. Results are from at least three independent experiments. Statistically significant differences are indicated: ^*^p < 0.05; ^**^p < 0.01, ^***^p < 0.001, and ^****^p < 0.0001.

Besides translation and protein stability, regulation at the transcriptional level represents another mechanism to control protein expression. We therefore assessed if K18 depletion had an impact on transcript levels of the different receptors by qRT-PCR on mRNAs extracted from control and K18-depleted HeLa cells. *cMet, TfR*, and *integrin* β*1* mRNA levels were strongly decreased in K18-depleted cells (Figure 8A), with reductions ranging from 54% for *cMet* to up to 94% for *TfR*. Such reduced mRNA levels should therefore be responsible for the reduced cMet, TfR, and integrin β1 protein levels detected in K18-depleted cells.

Decreased steady state mRNA levels may result from a reduction in transcription or from higher instability of the mRNA (Wu and Brewer, 2012). To assess the involvement of K18 in the stability of *cMet, TfR*, and *integrin* β*1* transcripts, we measured mRNA decay in cells treated with the transcription inhibitor Actinomycin D. Control and K18-depleted HeLa cells were left untreated (0 h) or incubated with Actinomycin D for 1 and 2 h, total RNAs were extracted and analyzed by qRT-PCR. We observed that *cMet, TfR*, and *integrin* β*1* mRNAs consistently displayed a higher rate of decay in K18-depleted cells (Figure 8B), thus, indicating higher instability of these transcripts in cells lacking K18.

Taken together, these results demonstrate that K18 confers stability to specific transmembrane receptor mRNAs thus ensuring steady state protein levels.

## Discussion

Manipulation of the host cell cytoskeleton is a hallmark of the cellular infection by several human bacterial pathogens. Intermediate filaments were reported to participate in the infection process of different pathogens (Geisler and Leube, 2016), however the molecular details remain sparse. Here we demonstrate for the first time that epithelial K8 and K18 play a dual role during *L. monocytogenes* cellular infection. We found that K8 and K18 are specifically required for the successful InlB/cMet-mediated *L. monocytogenes* cell invasion by modulating the actin dynamics at the entry site and by controlling the expression of *cMet* itself. Interestingly, K18 also appeared to control the expression of other cell surface receptors, such as TfR and integrin β1, by promoting mRNA stability, thus suggesting a broader role for keratins in the regulation of gene expression.

During infection, K8 and/or K18 were previously shown to assist toxin internalization (Nava-Acosta and Navarro-Garcia, 2013), to favor intracellular pathogen replication (Claser et al., 2008) and to allow stable pathogen docking to the host cell surface (Carlson et al., 2002; Batchelor et al., 2004; Russo et al., 2016). Moreover, K8 and K18 were shown to be targeted for degradation during viral and bacterial infections (Chen et al., 1993; Seipelt et al., 2000; Savijoki et al., 2008), however the functional details of these roles remain elusive.

Keratins, as other IFs, are dynamic filament networks that interact with a multitude of proteins serving as scaffolds to organize signaling platforms and regulate different processes (Pallari and Eriksson, 2006). How K8 and K18 modulate the actin dynamics during InlB-mediated cellular invasion is still unknown. Indeed, despite several reports pointing to an interplay between actin and keratin cytoskeletons, the molecular details of such a crosstalk remain largely unidentified (Jiu et al., 2015). The link between keratins and actin is thought to be mediated by their association with linker proteins such as plectin and dystrophin (Stone et al., 2005; Karashima et al., 2012). However, other IFs such as vimentin interact directly with actin or indirectly through motors protein like myosin IIB (Esue et al., 2006; Menko et al., 2014). Actin filaments were suggested to promote the assemble of keratin network (Windoffer et al., 2006; Kölsch et al., 2009) by favoring the retrograde transports of keratin subunits. Interestingly, the formation of EGF-induced actin-rich lamellipodia was shown to be followed by the extension of the keratin network and *de novo* nucleation at the lamellipodia itself (Felkl et al., 2012). K8 and 18 were reported to interact with Grb2 and Cbl (Robertson et al., 1997; Blagoev et al., 2003; Duan et al., 2012), proteins involved in cMet signaling and InlB-dependent entry of *L. monocytogenes* (Ireton et al., 1999). In addition, keratins were found to regulate the size and organization of lipid rafts (Gilbert et al., 2012, 2016), which serve as surface membrane platforms promoting clustering of signaling molecules (Pizarro-Cerdá and Cossart, 2009), and whose integrity is required for successful InlB-mediated *L. monocytogenes* infection (Seveau et al., 2004). It is thus possible that, through interaction with adaptor proteins downstream the activation of cMet at specific places at the host plasma membrane, K8 and K18 may modulate actin dynamics at InlB entry sites. The identification of host proteins interacting with K8 and K18 specifically upon *L. monocytogenes* infection or canonical HGF-induced cMet activation should uncover the molecular details of keratin-mediated actin dynamics modulation.

Strikingly, our data highlight the role of K18 in the control of the expression of several cell surface receptors such as cMet, TfR and integrin β1. These findings are in agreement with a growing body of evidence that suggests that keratins regulate gene expression and translation (Asghar et al., 2016). Indeed, mice that lack type I or type II keratins display perturbed transcription (Kumar et al., 2015, 2016) and impaired protein expression (Vijayaraj et al., 2009). Keratin 17 was recently reported to be present in the nucleus where it interacts with the promoter regions of cytokine genes and the transcriptional regulator AIRE (Hobbs et al., 2015) thus regulating inflammatory response. Additionally, K17 regulates the shuttling between the nucleus and the cytoplasm of proteins such as hnRNP K (Chung et al., 2015), 14-3-3σ (Kim et al., 2006), and p27^KIP1^ (Escobar-Hoyos et al., 2015). Nuclear accumulation of non-filamentous K18 was detected when exportin1-mediated nuclear export is inhibited (Kumeta et al., 2013), suggesting that K18, among others, may assist the nucleocytoplasmic shutting of proteins.

These observations, together with our data showing that K18 ensures the stability of certain mRNAs and thus promotes the expression of proper protein levels, tempt us to speculate that K18 may affect the shuttling of RNA-binding proteins (RBPs) from the nucleus to the cytoplasmic compartment, or the binding of specific RBPs involved in mRNA stabilization, and thus impact mRNA stability. In support to this hypothesis, K18 was shown to interact with hnRNP R (Havugimana et al., 2012), an RBP that binds and stabilizes the mRNA of MHC class I genes, thus enhancing their translation (Reches et al., 2016). In addition, while searching for K18 interactors (our unpublished data), we identified by mass spectrometry the heat-shock cognate protein 70 (Hsc70), a chaperone that is able to bind and stabilize the mRNA of the proapoptotic protein Bim (Matsui et al., 2007). We also identified the PTB-associated splicing factor (PSF), an RNA and DNA binding protein that regulates transcription, alternative splicing and mRNA stability (Yarosh et al., 2015). Finally, K18 was reported to interact with the mRNA degradation machinery protein Pan2 (Bett et al., 2013), involved in the initial trimming of polyadenylated tails of mRNA, a process that favors further mRNA deadenylation and subsequent degradation (Wu and Brewer, 2012). Together with K18, knockout of K8 results in perturbed mRNA levels of multiple genes (Habtezion et al., 2011; Asghar et al., 2016; Lähdeniemi et al., 2017).

Grounded in these previous studies and our data, we propose here that K18 might modulate the stability of particular transcripts probably by interacting with specific RBPs in the cytoplasm, thus modulating the fate of the associated transcripts and ultimately controlling gene expression. The molecular understanding of the role of K18 in mRNA stability and protein expression requires further studies to identify putative RBPs interacting with K18.

## Author contributions

RC, DC, and SS: conceived and designed the experiments; RC and MA: performed the experiments; RC, IP-C, AM, DC, and SS: analyzed the data; RC, DC, and SS: wrote the manuscript.

### Conflict of interest statement

The authors declare that the research was conducted in the absence of any commercial or financial relationships that could be construed as a potential conflict of interest.

## Supplementary material

Supplemental Figure 1 Keratin 8 (K8) and Keratin 18 (K18) are dispensable for *Listeria* infection of Caco-2 cells. Intracellular levels of *L. monocytogenes* were assessed by gentamicin protection assay and CFU counting in intestinal epithelial cell line Caco-2 cells that were left untransfected (NT) or transfected with control siRNA (Ctr) or with siRNAs specifically targeting K8 (K8-si, left panel), K18 (K18-si, middle panel) or both (K8/K18-si, right panel). The number of intracellular *L. monocytogenes* in NT cells was normalized to 100%, and those in siRNA-transfected cells were expressed as relative values to NT cells. Values are the mean ±S.E. of at least three independent experiments, each done in triplicate.

Supplemental Figure 2 K8 and K18 depletion efficiency in HeLa and Caco-2 cells. Efficiency of protein knockdown in **(a)** HeLa and **(b)** Caco-2 cells was assessed by western immunoblot using GAPDH as loading control. **(c)** Immunofluorescence images of Ctr and K8- (K8-si) or K18- (K18-si) depleted HeLa cells labeled for K8 and K18. Signal intensity was quantified. The values in Ctr cells were normalized to 1, and those in K8- and K18-depleted cells were expressed as relative values. Values are the mean ± S.E. of three independent experiments.

Supplemental Figure 3 K8 and K18 are not important for *Listeria* intracellular replication in HeLa cells. **(a)** Intracellular replication of *L. monocytogenes* in HeLa cells left untransfected (NT) or transfected with control (Ctr) or both K8 and K18 siRNA (K8/K18-si). Values represent the mean of duplicate samples from one representative experiment out of two independent experiments. **(b)** Efficiency of protein knockdown was assessed by western blot using GAPDH as loading control.

Supplemental Figure 4 K8 and K18 assist actin depolymerization during InlB-mediated internalization. Quantification of InlB-coated latex beads associated to polymerized actin in HeLa cells transfected with control (Ctr) or different concentrations of specific siRNA targeting K8 (K8-si) or K18 (K18-si). The use of 46 nM siRNA allows the maximum keratin depletion while 13.8 nM allows partial depletion. Cells were incubated with InlB-coated latex beads for 15, 30 and 60 min, fixed and stained for F-actin. Beads displaying actin recruitment were considered recruitment-positive. The total number of beads associated to cells was determined in brightfield. Values represent the mean ±S.E. of two independent experiments.

Supplemental Figure 5 K18 depletion perturbs expression and surface localization of transmembrane receptors in Caco-2 cells. Biotinylated surface proteins of control (Ctr) and K18-depleted (K18-si) Caco-2 cells were recovered from total cell extracts and pulled down using neutravidin beads. Biotinylated samples and whole cell lysates (WCL) were immunoblotted to detect cMet, TfR and integrin β1. **(a)** Immunoblot representative of two independent experiments. **(b)** Quantifications of E-cadherin, cMet, TfR and integrin β1 in WCL and in biotinylated samples from two independent experiments.
