# Supplementary figures and images for: Epithelial Keratins Modulate cMet Expression and Signaling and Promote InlB-Mediated Listeria monocytogenes Infection of HeLa Cells
Source: Front Cell Infect Microbiol. 2018 May 14;8:146. doi: 10.3389/fcimb.2018.00146 (PMC5960701; doi:10.3389/fcimb.2018.00146)

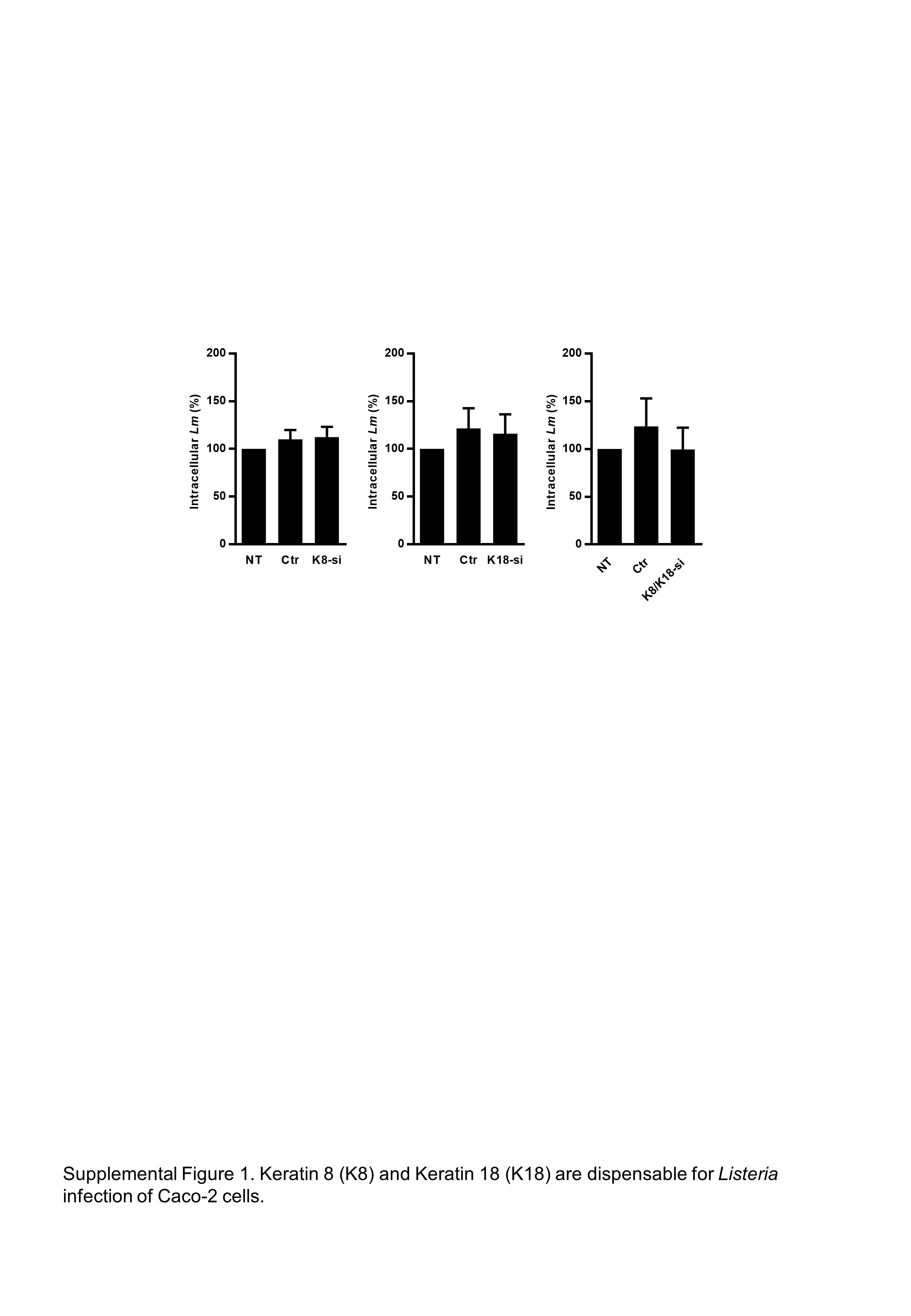

Supplement: Supplemental Figure 1 — Keratin 8 (K8) and Keratin 18 (K18) are dispensable for Listeria infection of Caco-2 cells. Intracellular levels of L. monocytogenes were assessed by gentamicin protection assay and CFU counting in intestinal epithelial cell line Caco-2 cells that were left untransfected (NT) or transfected with control siRNA (Ctr) or with siRNAs specifically targeting K8 (K8-si, left panel), K18 (K18-si, middle panel) or both (K8/K18-si, right panel). The number of intracellular L. monocytogenes in NT cells was normalized to 100%, and those in siRNA-transfected cells were expressed as relative values to NT cells. Values are the mean ±S.E. of at least three independent experiments, each done in triplicate. [file Image_1.TIF]

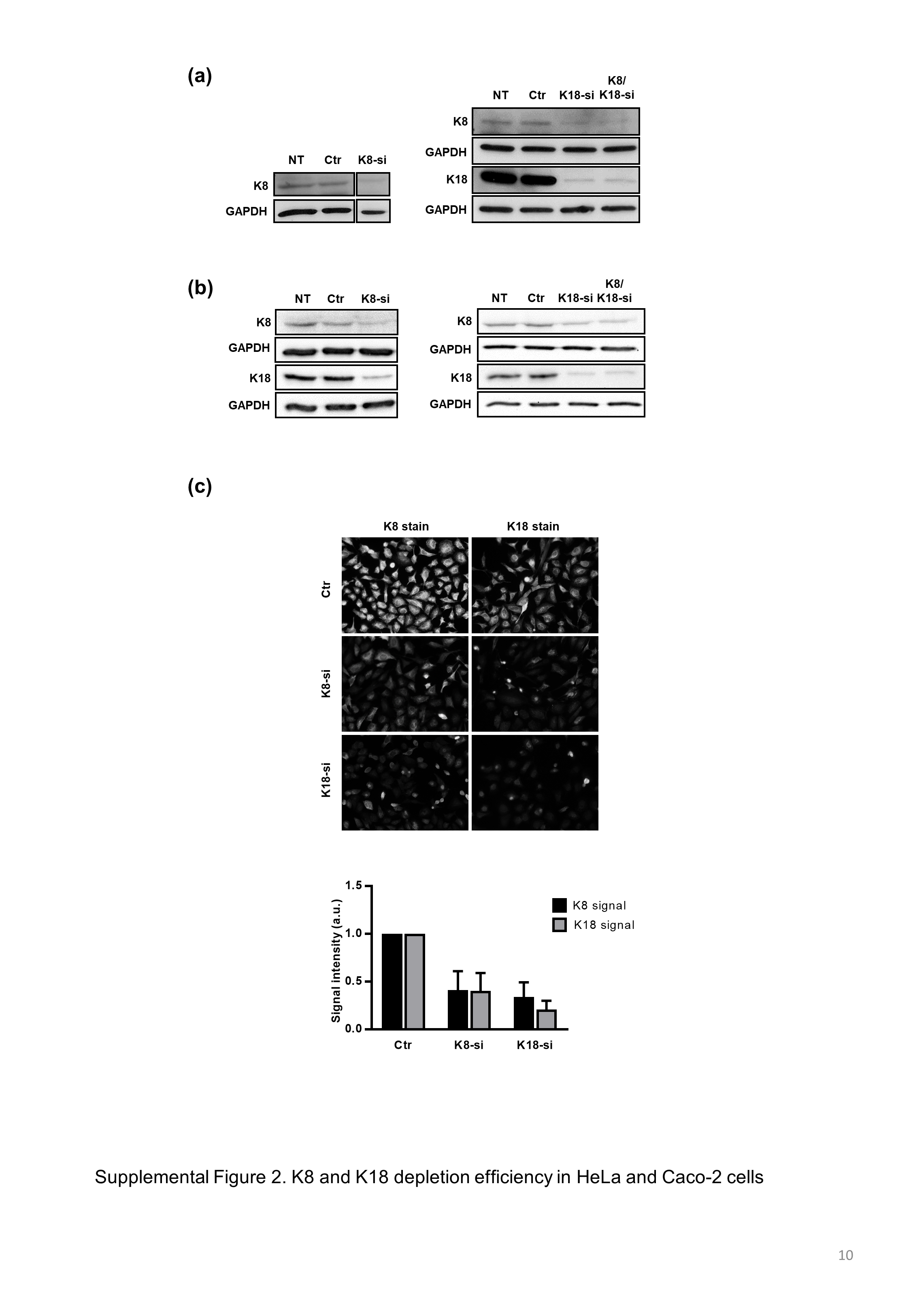

Supplement: Supplemental Figure 2 — K8 and K18 depletion efficiency in HeLa and Caco-2 cells. Efficiency of protein knockdown in (a) HeLa and (b) Caco-2 cells was assessed by western immunoblot using GAPDH as loading control. (c) Immunofluorescence images of Ctr and K8- (K8-si) or K18- (K18-si) depleted HeLa cells labeled for K8 and K18. Signal intensity was quantified. The values in Ctr cells were normalized to 1, and those in K8- and K18-depleted cells were expressed as relative values. Values are the mean ± S.E. of three independent experiments. [file Image_2.TIF]

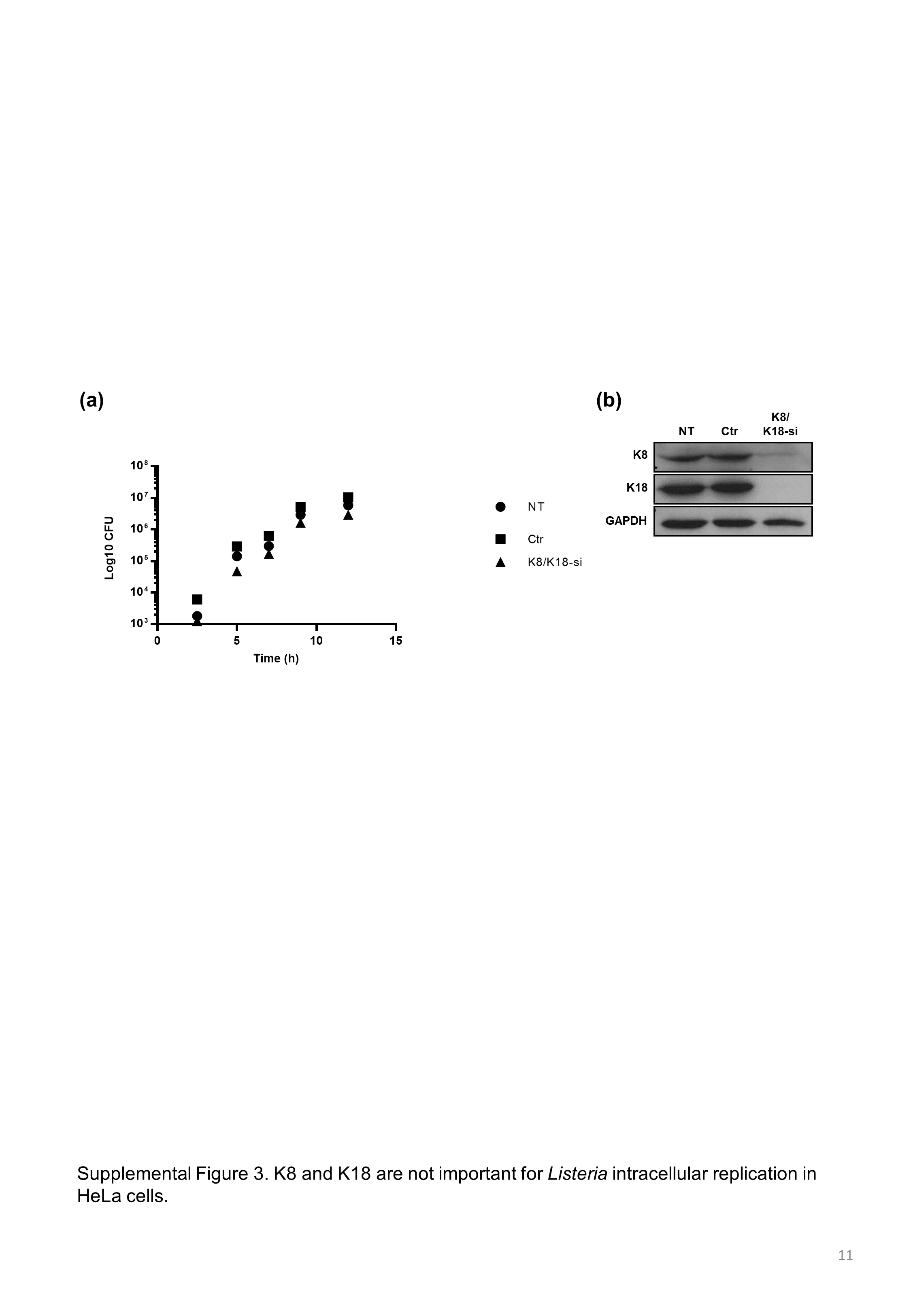

Supplement: Supplemental Figure 3 — K8 and K18 are not important for Listeria intracellular replication in HeLa cells. (a) Intracellular replication of L. monocytogenes in HeLa cells left untransfected (NT) or transfected with control (Ctr) or both K8 and K18 siRNA (K8/K18-si). Values represent the mean of duplicate samples from one representative experiment out of two independent experiments. (b) Efficiency of protein knockdown was assessed by western blot using GAPDH as loading control. [file Image_3.TIF]

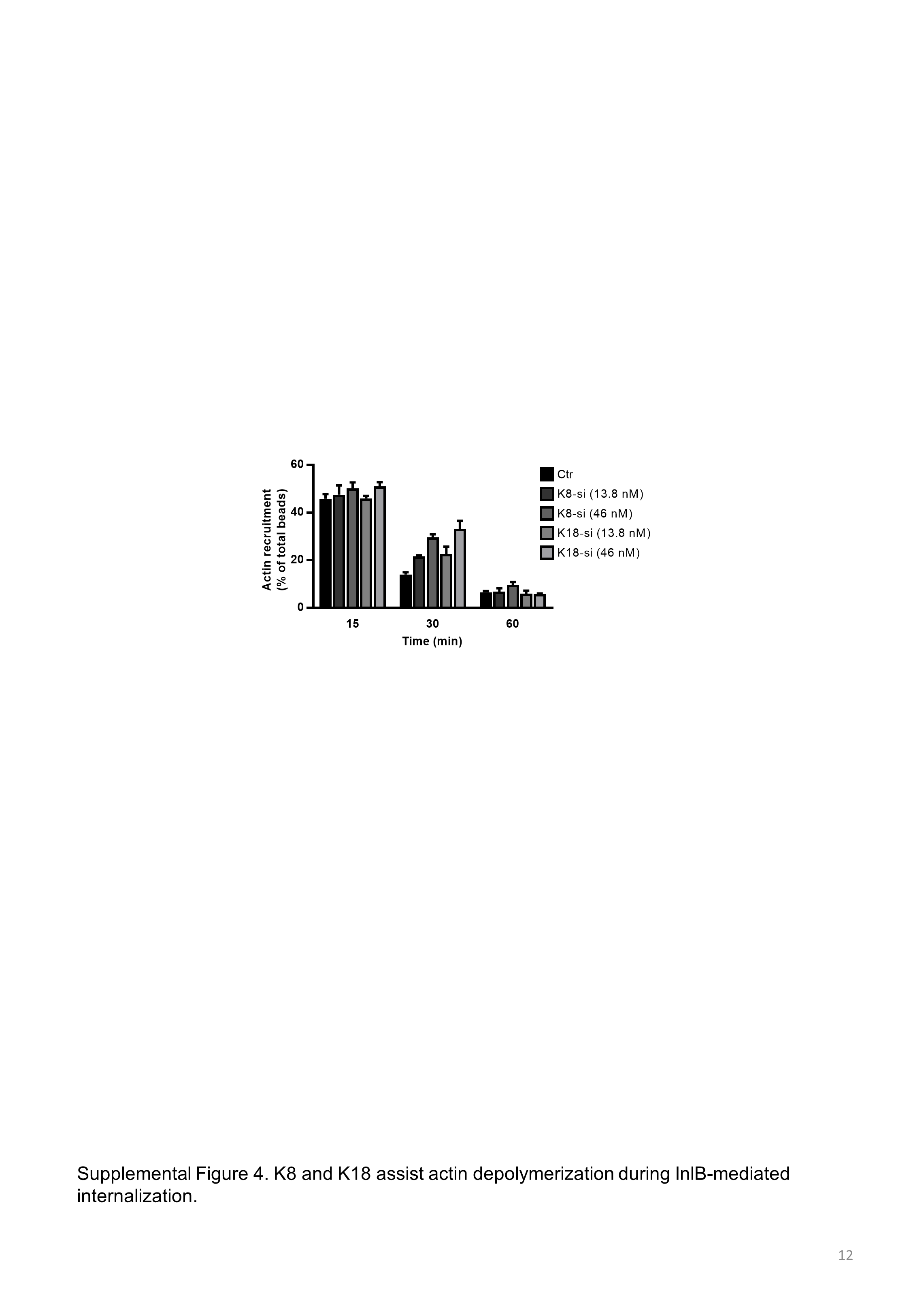

Supplement: Supplemental Figure 4 — K8 and K18 assist actin depolymerization during InlB-mediated internalization. Quantification of InlB-coated latex beads associated to polymerized actin in HeLa cells transfected with control (Ctr) or different concentrations of specific siRNA targeting K8 (K8-si) or K18 (K18-si). The use of 46 nM siRNA allows the maximum keratin depletion while 13.8 nM allows partial depletion. Cells were incubated with InlB-coated latex beads for 15, 30 and 60 min, fixed and stained for F-actin. Beads displaying actin recruitment were considered recruitment-positive. The total number of beads associated to cells was determined in brightfield. Values represent the mean ±S.E. of two independent experiments. [file Image_4.TIF]

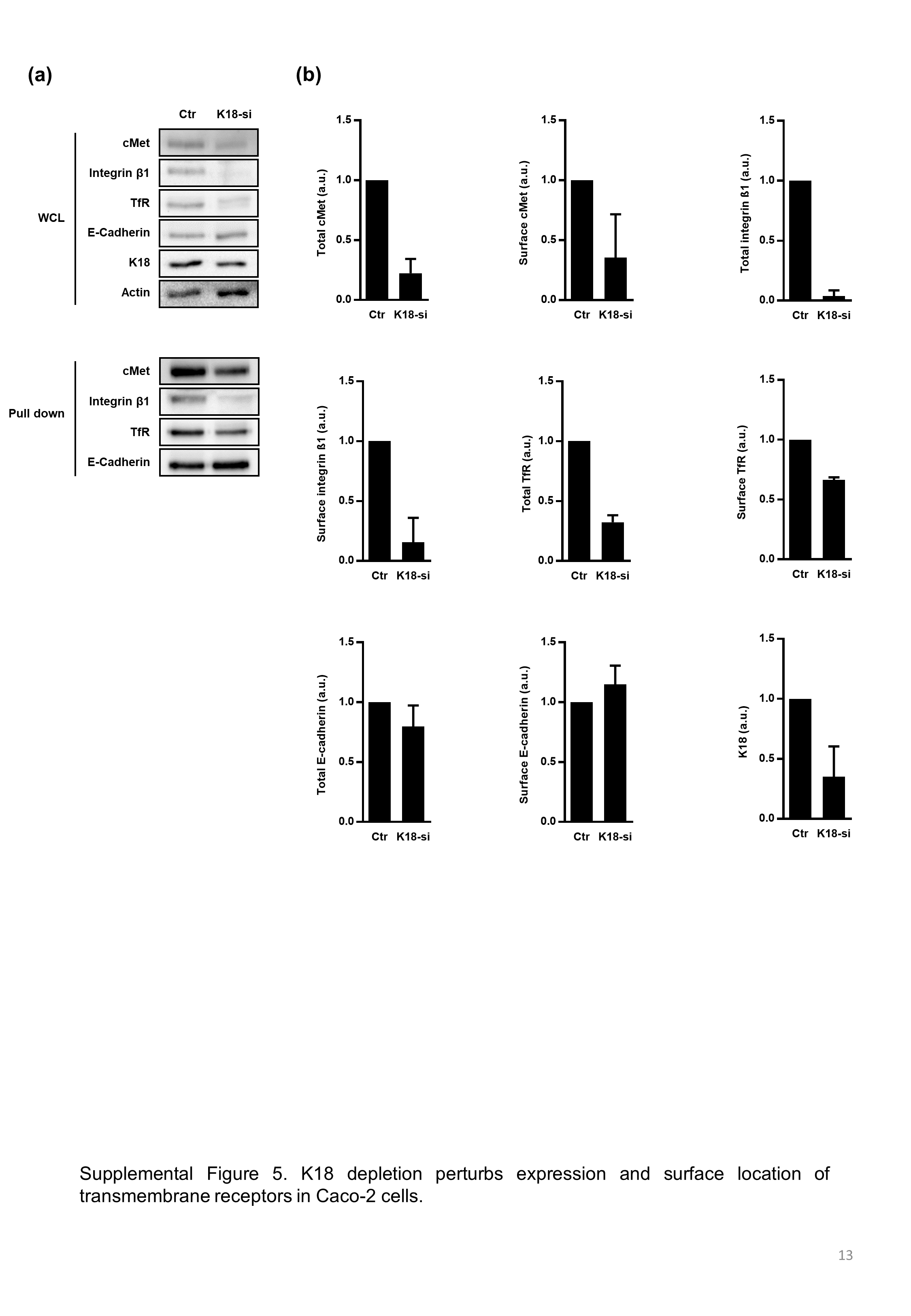

Supplement: Supplemental Figure 5 — K18 depletion perturbs expression and surface localization of transmembrane receptors in Caco-2 cells. Biotinylated surface proteins of control (Ctr) and K18-depleted (K18-si) Caco-2 cells were recovered from total cell extracts and pulled down using neutravidin beads. Biotinylated samples and whole cell lysates (WCL) were immunoblotted to detect cMet, TfR and integrin β1. (a) Immunoblot representative of two independent experiments. (b) Quantifications of E-cadherin, cMet, TfR and integrin β1 in WCL and in biotinylated samples from two independent experiments. [file Image_5.TIF]
